# The Enhanced Red Emission and Improved Thermal Stability of CaAlSiN_3_:Eu^2+^ Phosphors by Using Nano-EuB_6_ as Raw Material

**DOI:** 10.3390/nano8020066

**Published:** 2018-01-25

**Authors:** Wen-Quan Liu, Dan Wu, Hugejile Chang, Ru-Xia Duan, Wen-Jie Wu, Guleng Amu, Ke-Fu Chao, Fu-Quan Bao, Ojiyed Tegus

**Affiliations:** 1 Inner Mongolia Key Laboratory for Physics and Chemistry of Functional Materials, College of Physics and Electronic Information, Inner Mongolia Normal University, Hohhot 010022, China; seazuri@163.com (W.-Q.L.); criffchang@gmail.com (H.C.); ruxia1028@163.com (R.-X.D.); 15248127843@163.com (W.-J.W.); b.fuquan@imnu.edu.cn (F.-Q.B.); 2 School of Physical Science and Technology, & Inner Mongolia Key Lab of Nanoscience and Nanotechnology, Inner Mongolia University, Hohhot 010021, China; wudan20101203@163.com; 3 Department of physics and electrical engineering, Xilingol Vocational College, Xilinhot 0026000, China; amgl15947298308@163.com

**Keywords:** alloy precursors, nitride, CaAlSiN_3_:Eu^2+^red phosphor, nano-EuB_6_

## Abstract

Synthesizing phosphors with high performance is still a necessary work for phosphor-converted white light-emitting diodes (W-LEDs). In this paper, three series of CaAlSiN_3_:Eu^2+^ (denoted as CASN:Eu^2+^) phosphors using Eu_2_O_3_, EuN and EuB_6_ as raw materials respectively are fabricated by under the alloy precursor normal pressure nitridation synthesis condition. We demonstrate that CASN:Eu^2+^ using nano-EuB_6_ as raw material shows higher emission intensity than others, which is ascribed to the increment of Eu^2+^ ionic content entering into the crystal lattice. An improved thermal stability can also be obtained by using nano-EuB_6_ due to the structurally stable status, which is assigned to the partial substitution of Eu–O (Eu–N) bonds by more covalent Eu–B ones that leads to a higher structural rigidity. In addition, the W-LEDs lamp was fabricated to explore its possible application in W-LEDs based on blue LEDs. Our results indicate that using EuB_6_ as raw materials can provide an effective way of enhancing the red emission and improving the thermal stability of the CASN:Eu^2+^ red phosphor.

## 1. Introduction

White light-emitting diodes (W-LEDs) are considered as the next-generation solid-state lighting system due to the high luminous efficiency, long operation time, reliability environmental friendliness [1,2,3]. The traditional way used to obtain white light is combing blue InGaN LEDs chip with the yellow phosphor Y_3_Al_5_O_12_:Ce^3+^ (YAG:Ce^3+^). However, using a single yellow phosphor leads to low color rendering index (Ra < 80) due to the insufficient red component in the spectra, making them unsuitable for high-quality “warmer” white lighting [4,5,6,7]. Therefore, a red emitting phosphor is thus essentially needed to enhance the red spectral part to achieve much higher color rendering indices.

Currently, several red emitting phosphors attract much attention and are pervasively studied due to the property of effectively enhancing the color rendition, such as M_2_Si_5_N_8_:Eu^2+^ (M = Ca, Ba, Sr) [8,9,10,11], SrLiAl_3_N_4_:Eu^2+^ [12,13,14,15] and CASN:Eu^2+^ [16,17,18,19]. Among the above red phosphors, the emission of Eu^2+^ in CaAlSiN_3_ host locates in deep red region and possesses the advantages of good thermal stability, reliability and high quantum efficiency, which make it superior to others. There are many preparation methods in fabricating CASN:Eu^2+^ red phosphors each has its own shortcomings. Commonly, the metal and rare earth nitrides are used as raw materials in preparing CASN:Eu^2+^ red phosphors they have to be operated in glove box because nitride compounds are unstable, decomposable and oxidizable in the air hence, the ideas of inhibiting certain disintegrating process put higher requirements to the experimental equipment and additional standards of higher temperature (≥1800°) and pressure (≥1 MPa) [20,21]. For example, experimental expensive and yield small amounts of outcome when it comes to spark plasma sintering (SPS) process [22,23] and self-propagating high temperature synthesis (SHS) procedures [19,24], therefore, it is difficult to be carried out in manufacturing. In contrast, the alloy precursor normal pressure nitridation process is easier to operate rested upon high active alloy without high temperature (≥1800°) and high pressure (≥1 MPa) requirement, whereas the oxidation of the raw material is difficult to avoid. Higher oxygen content in raw materials necessitates it to be processed with oxidation-reduction treatment. To this end, several very simple methods like gas reduction nitridation (GRN) method [25,26] and carbothermal reduction and nitridation (CTRN) method [27,28] are applied to the preparation of CASN:Eu^2+^ nitride materials. However, the obtained product contained impurities of unreacted CaO and excess C powders. Combined with, alloy precursor and non-oxygen raw materials will help improve luminescence performance of CASN:Eu^2+^ series nitride phosphors.

In this paper, a high performance CASN:Eu^2+^ red phosphors, using nano-EuB_6_ as raw materials, were obtained by alloy precursor normal pressure nitridation. The above red phosphor exhibits an enhanced red emission and improved thermal stability, which permits it to be superior to the common used CASN:Eu^2+^ phosphors using Eu_2_O_3_ and EuN as raw materials. The possible performance of the red phosphor in W-LED was also investigated.

## 2. Experimental 

### 2.1. Sample Preparation

The powder samples were synthesized by the alloy precursor normal pressure nitridation method in a high temperature tubular furnace with the raw materials of CaSi alloy, AlN (99.5%), Eu_2_O_3_ (99.99%), EuN (99.9%) and nano-EuB_6_. EuB_6_ is a typical semimetal material, Eu atoms are centered as an octahedron composed of six rigidly bound lighter atoms of boron. Thus, it has rich structure and excellent physical and chemical properties [29,30]. The energy bands of the EuB_6_ can be considered to be formed by orbital interactions between Eu^2+^ and B_6_^2−^ clusters. Synthesized nano-EuB_6_ was reported method by the Bao et al. [31]. Eu_2_O_3_ (99.99%) and NaBH_4_ (99.0%) mixed in an agate mortar for 30 min. Then the mixtures were put into a quartz tube and placed in the resistance furnace at a reaction temperature in the range from 1150 °C to 1200 °C for 2 h. Therein CaSi alloy is sintered by reactants Ca (99.5%), Si (99.999%) through arc melting process in Ar gas. The resulting mixtures that using Eu_2_O_3_ and EuN as raw materials were heated at 1550 °C for 4 h under a 200 mL min^−1^ N_2_ (99.999%) and 20 mL min^−1^ H_2_ (99.999%) reducing atmosphere with a constant flow rate 100 mL/min while that using nano-EuB_6_ were heated at 1550 °C for 4 hundred a 200 mL min^−1^ N_2_ (99.999%). Finally, the samples were cooled to room temperature in the furnace and ground again for the following characterization.

### 2.2. Measurements and Characterization

The microstructure and chemical composition of powders were imaged and measured by using a scanning electron microscopy (SEM, Hitachi S-3400N, Hitachi High-Tech Fielding Corporation, Tokyo, Japan) and X-ray energy dispersive spectrometer (EDS, Hitachi, Tokyo, Japan) equipment. The crystal structure was determined via X-ray powder diffraction (XRD, Philip PW1830 with Cu Kα radiation operating at 30 kV and 30 mA, Amsterdam, The Netherlands) at room temperature. The X-ray photoelectron spectroscopy (XPS) spectra were carried out with the X-ray photoelectron spectrometer (ESCALAB 250, Thermo Fisher Scientific, Loughborough, UK) using Al Kα_1_ radiation at 15 kV and 10 mA. The emission spectra were measured by a Hitachi F4600 fluorescence spectrophotometer (Hitachi, Tokyo, Japan). The temperature-dependent PL spectra were also carried out on Hitachi F-7000 spectrometer (Hitachi, Tokyo, Japan) with an external heater. 

A white LED was fabricated by combining a blue-LED chip (455 nm) with commercial green phosphor G3537, the as-synthesized CASN:Eu^2+^. The optical properties of the fabricated W-LEDs were measured using sphere spectroradiometer system (LHS-1000, Everfine Co., Hangzhou, China). The working bias voltage and current of LEDs are respectively 3.4 V and 20 mA. All the measurements were conducted at room temperature unless mentioned specially. 

## 3. Results and Discussion

### 3.1. Structure Characterization

**Figure 1:**
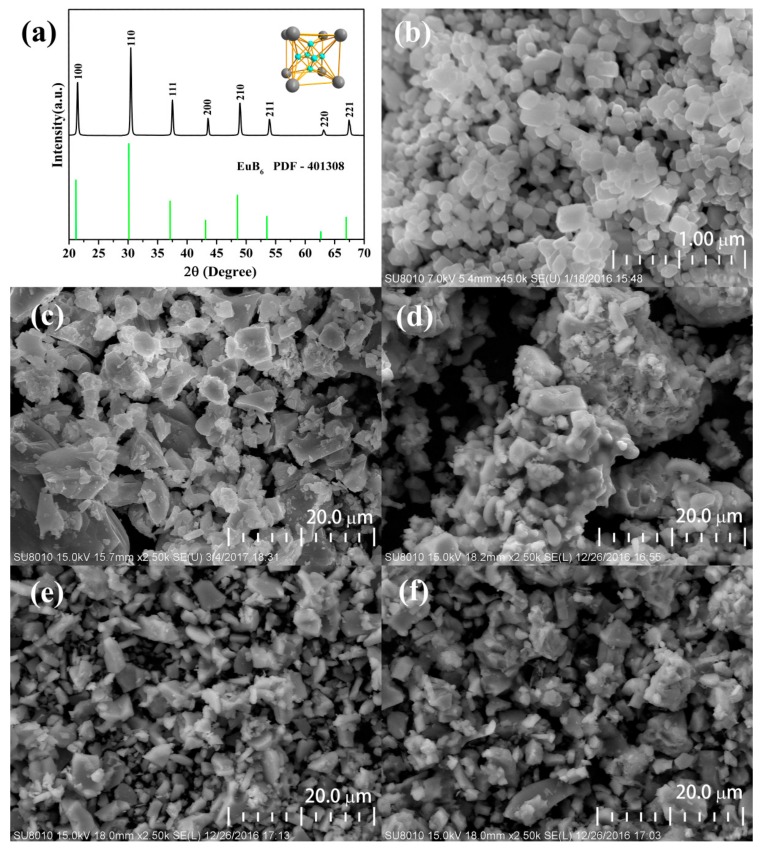
XRD patterns of (**a**) nano-EuB_6_ and the SEM images of (**b**) nano-EuB_6_; (**c**) CaSi alloys; (**d**) Ca_0.94_AlSiN_3_:0.06Eu^2+^@Eu_2_O_3_; (**e**) Ca_0.94_AlSiN_3_:0.06Eu^2+^@EuN; and (**f**) Ca_0.94_AlSiN_3_:0.06Eu^2+^@EuB_6_.

The XRD pattern and the crystal structure of the used nano-EuB_6_ were shown in Figure 1a. The morphologies of nano-EuB_6_, CaSi alloy powder and the as-synthesized samples CASN:Eu^2+^@Eu_2_O_3_, CASN:Eu^2+^@EuN, as well as CASN:Eu^2+^@EuB_6_ were observed by the SEM image in Figure 1b–f. The central particle size of the used nano-EuB_6_ is around 200~300 nm. One can find that there exists an obvious agglomeration in CASN:Eu^2+^@Eu_2_O_3_, whose particles is relatively bigger and irregular than those of CASN:Eu^2+^@EuN and CASN:Eu^2+^@EuB_6_.

The EDS analytical data of CaSi alloys, CASN:Eu^2+^@Eu_2_O_3_, CASN:Eu^2+^@EuN and CASN:Eu^2+^@EuB_6_ were listed in Table 1. One can find that there exists a small amount of oxygen in CaSi alloys and it cannot be avoided effectively. The EDS analysis reveals that CASN:Eu^2+^@EuB_6_ contains least oxygen while the maximum oxygen content in CASN:Eu^2+^@Eu_2_O_3_. The possible explanation of this phenomenon is that Eu_2_O_3_ is not fully reduced by local substances during sintering operations. It should be mentioned that the powders synthesized by EuN as the raw material are easier to be oxidized due to its high activity. Comparatively, the EuB_6_ has the properties of physically and chemically stable, which prevents it from oxidation. The small amount of oxygen impurities in CASN:Eu^2+^@EuB_6_ are also attributed to the CaSi alloy, which could be reduced under a reducing atmosphere.

**Figure 2:**
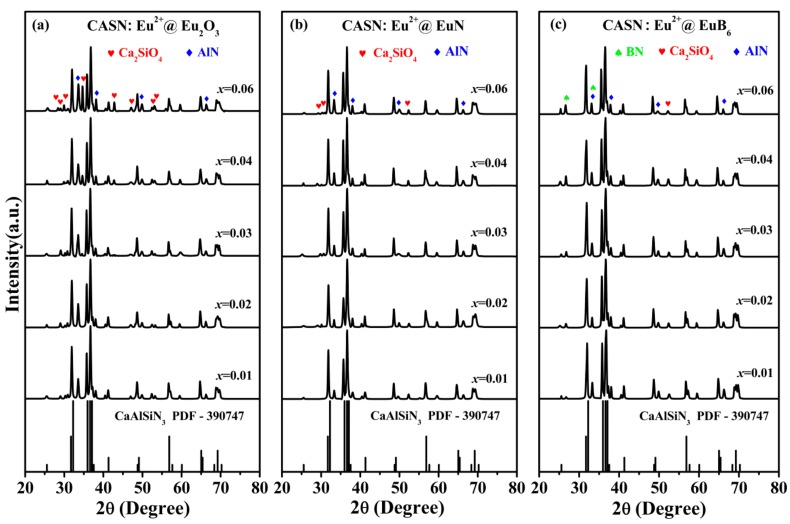
XRD patterns of three nitride red phosphors using different raw materials (**a**) CASN:Eu^2+^@Eu_2_O_3_; (**b**) CASN:Eu^2+^@EuN and (**c**) CASN:Eu^2+^@EuB_6_.

**Figure 3:**
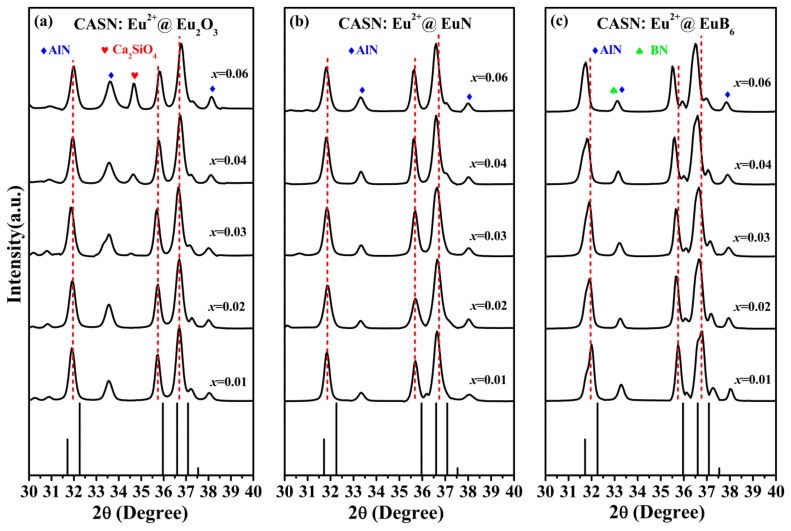
The major XRD patterns of three nitride red phosphors in the range of 30–40°; (**a**) CASN:Eu^2+^@Eu_2_O_3_; (**b**) CASN:Eu^2+^@EuN and (**c**) CASN:Eu^2+^@EuB_6_.

The XRD patterns of three series CASN:Eu^2+^ (*x* = 0.01, 0.02, 0.03,0.04, 0.06) phosphors using Eu_2_O_3_, EuN and EuB_6_ as raw materials, labeled as CASN:Eu^2+^@Eu_2_O_3_, CASN:Eu^2+^@EuN as well as CASN:Eu^2+^@EuB_6_ respectively, are shown in Figure 2 the standard XRD pattern of CaAlSiN_3_ (PDF-390747) is also shown for comparison. As illustrated in Figure 2, the diffraction peaks are predominantly identified as CaAlSiN_3_ phase and crystallize in the orthorhombic space group Ccm2_1_. It should be noted that the impurity phases, Ca_2_SiO_4_ (PDF-110585) and AlN (PDF-871054) in CASN:Eu^2+^@Eu_2_O_3_ and CASN:Eu^2+^@EuN as well as an extra phase BN (PDF-090012) in CASN:Eu^2+^@EuB_6_ are also identified, as shown in Figure 2a–c. Since the synthesis conditions of CASN:Eu^2+^ phosphors need a high requirement for installations, the impurity phases are unavoidable during the sinter process. In our work, the impurity phase Ca_2_SiO_4_ emerged during the preparation of CaSi alloy due to the oxidized process and it is expected to increase with the appearance of a rich oxygen source Eu_2_O_3_, which is confirmed by the more intense peak intensity in CASN:Eu^2+^@Eu_2_O_3_ than that in CASN:Eu^2+^@EuN and CASN:Eu^2+^@EuB_6_. The above result is also consistent with the ratios of N/O in different samples that measured by EDS (see Table 1). For the impurity phase AlN, its precise stoichiometric ratio in the reaction can hardly be determined due to its low solubility in CaAlSiN_3_, which results in the redundant AlN [32,33]. It can be found from Figure 3 that the main diffraction peaks of CaAlSiN_3_ in the range of 30–40° shift to a low degree with the increase of Eu^2+^ ion concentration in CASN:Eu^2+^@EuB_6_, which could be ascribed to be the substitution of larger B^2−^ (1.40 Å, CN = 4) for smaller N^3−^ (1.32 Å, CN = 4) [17]. In CASN:Eu^2+^@EuB_6_, the diffraction peaks at 2*θ* = 33.5° is superposed by AlN and BN, which are formed within the reaction between B and N_2_ (or AlN).

### 3.2. XPS Analysis

**Figure 4:**
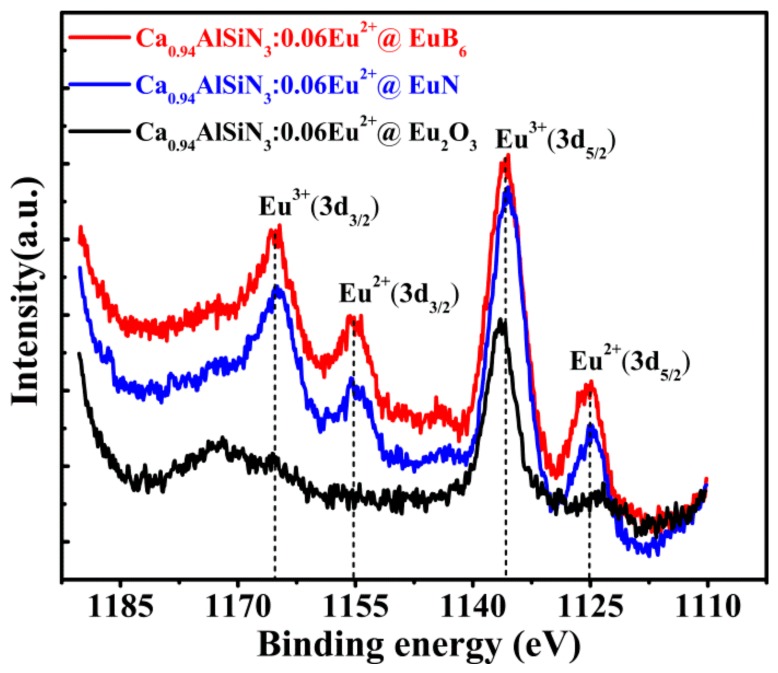
The XPS spectra of Eu_3*d*_ in Ca_0.94_AlSiN_3_:0.06Eu^2+^@Eu_2_O_3_ (black line), Ca_0.94_AlSiN_3_:0.06Eu^2+^@EuN (blue line) and Ca_0.94_AlSiN_3_: 0.06 Eu^2+^@EuB_6_ (red line).

The XPS measurement was conducted to study the local valence state of each element. The XPS spectra of Eu_3*d*_ in CASN:Eu^2+^@Eu_2_O_3_ (black line), CASN:Eu^2+^@EuN (blue line), as well as CASN:Eu^2+^@EuB_6_ (red line) were shown in Figure 4. According to the certain references [34,35], the bands peaking at 1165.27 eV and 1135.86 eV in Eu_3*d*_ XPS spectra correspond to Eu^3+^(3*d_3/2_*) and Eu^3+^(3*d_5/2_*) respectively, while that peaking at 1155.22 eV and 1125.02 eV are attributed to Eu^2+^(3*d_3/2_*) and Eu^2+^(3*d_5/2_*). In the samples of CASN:Eu^2+^@EuN and CASN:Eu^2+^@EuB_6_, there exist the bands of Eu^3+^(3*d_3/2_*), Eu^2+^(3*d_3/2_*), Eu^3+^(3*d_5/2_*), Eu^2+^(3*d_5/2_*), while in CASN:Eu^2+^@Eu_2_O_3_, the bands of Eu^3+^(3*d_5/2_*) dominate the spectrum. Comparative result confirms that the peak intensity ratio of Eu^2+^ and Eu^3+^ are identical with its content ratio in EuN and EuB_6_ doped (see Table 2). The observation shows that Eu^2+^ content in CASN:Eu^2+^@EuN or CASN:Eu^2+^@EuB_6_ is nearly the same and both of them are higher than that in CASN:Eu^2+^@Eu_2_O_3_.

**Figure 5:**
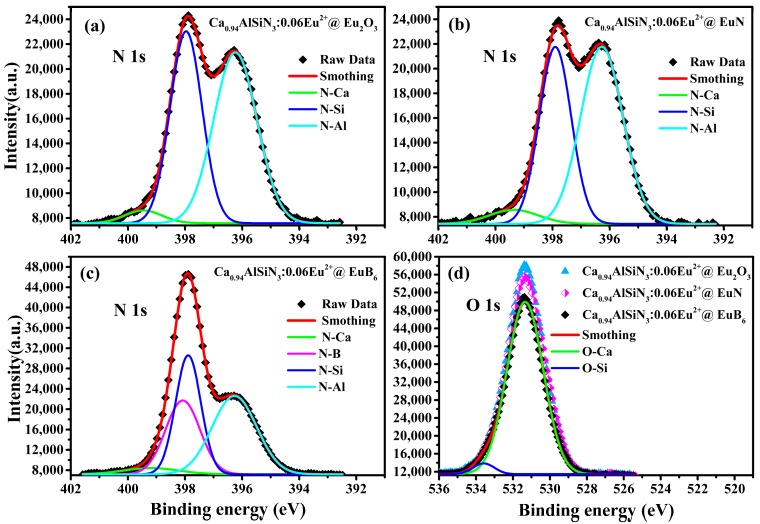
The XPS spectra of O_1s_ and N_1s_ Ca_0.94_AlSiN_3_:0.06Eu^2+^@Eu_2_O_3_, Ca_0.94_AlSiN_3_:0.06Eu^2+^@EuN and Ca_0.94_AlSiN_3_:0.06Eu^2+^@EuB_6._

Figure 5 shows the XPS spectra of N_1s_ and O_1s_ in CASN:Eu^2+^ samples series. The N_1s_ spectra, it can also be deconvoluted into two peaks: N–Ca (green line), N–Si (blue line) and N–Al (Cyan line) bonds. The content of N_1s_ that corresponds to N–Si bond in CASN:Eu^2+^@EuB_6_ is much higher than that in CASN:Eu^2+^@Eu_2_O_3_ and CASN:Eu^2+^@EuN at the same Eu doping level. It was reported that the binding energy of N_1s_ that correspond to N-B (magenta line) bond was around 397.6~398.5 eV [36,37]. One can find from the XRD data that there exists the impurity phase of BN in CASN:Eu^2+^@EuB_6_ sample. Therefore, it can be concluded that the peak intensity at the lower binding energy is the combined result of N–Si and N–B bonds. For the O_1s_ spectra can be deconvoluted into two peaks: O–Si (blue line) and O–Ca (green line) bonds. One can find that the peak intensity of the lower binding energy, which corresponds to O–Ca bond, has the minimum in CASN:Eu^2+^@EuB_6_ while has the maximum in CASN:Eu^2+^@Eu_2_O_3_. It can be concluded from the XRD data that the existence of the impurity phase of CaSiO_4_ is the main factor for the formation of O–Si and O–Ca bonds. Additionally, it can be found from the EDS results that oxygen content in CASN:Eu^2+^@EuB_6_ was significantly less than that in CASN:Eu^2+^@Eu_2_O_3_ and CASN:Eu^2+^@EuN.

### 3.3. Photoluminescence Properties

**Figure 6:**
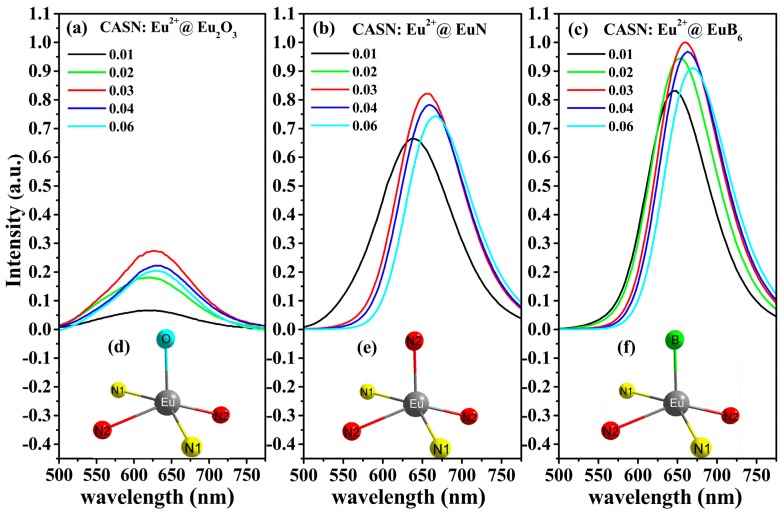
Photoluminescence spectra and crystallographic environments around Eu^2+^ ions of three series of nitride red phosphors using different raw materials (**a**) CASN:Eu^2+^@Eu_2_O_3_; (**b**) CASN:Eu^2+^@EuN; (**c**) CASN:Eu^2+^@EuB_6_; (**d**) EuN_2_^I^N_2_^II^O; (**e**) EuN_2_^I^N_3_^II^ and (**f**) EuN_2_^I^N_2_^II^B phosphors with various content *x* (*x* = 0.01, 0.02, 0.03, 0.04, 0.06).

The emission spectra of three groups of CASN:Eu^2+^@Eu_2_O_3_, CASN:Eu^2+^@EuN as well as CASN:Eu^2+^@EuB_6_ (*x* = 0.01, 0.02, 0.03, 0.04, 0.06) phosphors under 460 nm blue light excitation are shown in Figure 6. Upon the introduction of Eu^2+^ ion, the emission band attributed to the 5*d*→4*f* transition of Eu^2+^ ion dominates the spectra in all series of samples. One can find from Figure 6a–c that the emission intensity of Eu^2+^ ion in each group reaches its maximum value at *x* = 0.03, beyond which it starts to decrease due to the concentration quenching among Eu^2+^ ions. It should be noted that the emission intensity of Eu^2+^ ion in CASN:Eu^2+^@EuB_6_ is stronger than that in CASN:Eu^2+^@EuN and CASN:Eu^2+^@Eu_2_O_3_.

**Figure 7:**
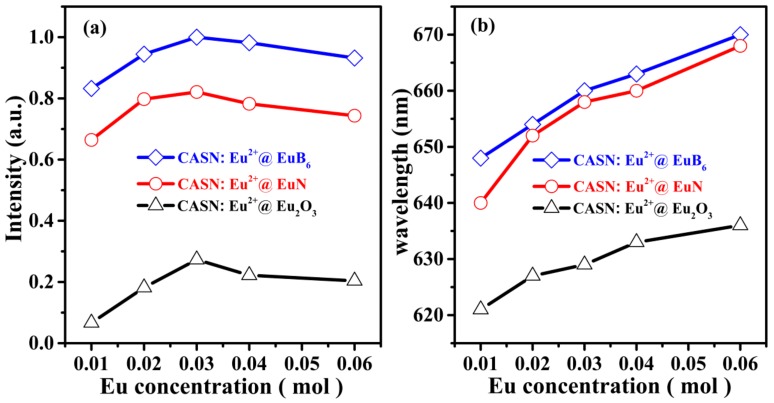
Dependence of emission intensity (**a**) and peak position (**b**) of CASN:Eu^2+^@Eu_2_O_3_ (black line), CASN:Eu^2+^@EuN (blue line) and CASN:Eu^2+^@EuB_6_ (red line) phosphors with various Eu^2+^ concentration (*x* = 0.01, 0.02, 0.03, 0.04, 0.06).

Under 460 nm blue light excitation, the emission peaks of CASN:Eu^2+^@Eu_2_O_3_ fall in the scope of 621~636 nm while that of CASN:Eu^2+^@EuN as well as CASN:Eu^2+^@EuB_6_ are in 640~668 nm, 648~670 nm, respectively. At the same Eu^2+^ doping concentration, the emission wavelength in CASN:Eu^2+^@EuB_6_ series is longer than that in CASN:Eu^2+^@EuN (as shown in Figure 7a,b). 

The XPS spectra reveal that Eu^2+^ content in both CASN:Eu^2+^@EuN and CASN:Eu^2+^@EuB_6_ is higher than that in CASN:Eu^2+^@Eu_2_O_3_. The amounts of O–Si, O–Ca bonds will decline accompanied by the increase of N-Si bond with the decrease of oxygen content in host materials, as a result, it affects the crystal field, which results in the split of Eu^2+^ energy levels. The position of the 5*d* emission band of the Eu^2+^ ions at lower energy (longer wavelength) is attributed to the influence of highly covalent bonding of Eu-*X* (*X* = N, B, O) and high crystal field strength. The reduction of crystal field strength around the Eu^2+^ ion in CASN:Eu^2+^@Eu_2_O_3_ leads to a blue shift. However, under the influence of crystal field, the emission spectra have a larger red shift in CASN:Eu^2+^@EuN and CASN:Eu^2+^@EuB_6_. This is caused by the shrinkage of 5*d*→4*f* energy level spacing [38]. 

Particularly, in the series of CASN:Eu^2+^@EuB_6_, with B^2−^ ions incorporating into the host lattice of CaAlSiN_3_, they substitute N^3−^ and O^2−^ ions through a pattern of EuN_2_^I^N_3_^II^→EuN_2_^I^N_2_^II^B (see Figure 6f) [39] then, a red shift in the spectrum was expected due to the changed crystal strength. Moreover, the Eu–N bond length critically affects the emission wavelength of nitride phosphors. Therefore, we believe that according to the above schematic pattern the length of the Eu–B bonds is longer than that of the Eu–N bonds (see Figure 6e,f). In addition, the metal cation ratio in CaAlSiN_3_ is Ca:Al:Si = 1:1:1, in which Ca and Al/Si occupied on the 4a and 8b sites in the space group of Ccm2_1_, respectively [25,32]. In the series of CASN:Eu^2+^@EuB_6_, the decrease of oxygen content can prevent the formation of Ca_2_SiO_4_ and increase the content of occupation Ca ions, which can also lead to the improvement of emission intensity. Since the three series of samples have the same Eu^2+^ doping level thus the higher luminescence performance of CASN:Eu^2+^@EuB_6_ indicates that Eu^2+^ ions content in the host lattice has changed the crystal environment and the site occupancy.

### 3.4. Thermal Stability Analysis

**Figure 8:**
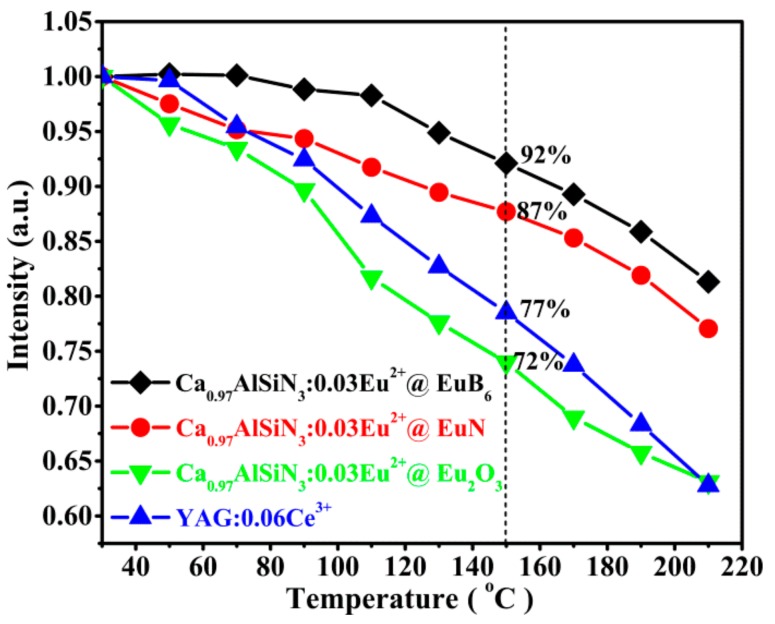
Temperature-dependent emission intensities of Ca_0.97_AlSiN_3_:0.03Eu^2+^@Eu_2_O_3_ (green line), Ca_0.97_AlSiN_3_:0.03Eu^2+^@EuN (red line), Ca_0.97_AlSiN_3_:0.03Eu^2+^@EuB_6_ (black line) and YAG:0.06Ce^3+^ (blue line) phosphors.

A stable emission intensity at the elevated temperature, typically at 150 °C or even higher for high-power application is a basic requirement for phosphors converted W-LEDs thus the phosphors must have small thermal quenching to maintain the long lifetime of LED devices. In order to evaluate the influence of the temperature on the luminescence, the temperature-dependent PL intensities of the as-prepared Ca_0.97_AlSiN_3_:0.03Eu^2+^@Eu_2_O_3_, Ca_0.97_AlSiN_3_:0.03Eu^2+^@EuN as well as Ca_0.97_AlSiN_3_:0.03Eu^2+^@EuB_6_ were given in Figure 8. The temperature–dependent PL intensities of YAG:0.06Ce^3+^ phosphor were also shown for comparison. It can be easily observed that all the above four phosphors exhibit thermal quenching in different degrees with temperature increasing from 30 °C to 210 °C. In addition, one can find that as the temperature increased to 150 °C, the emission intensity declined to 72%, 87%, 92% and 77% of their initial values at room temperature for Ca_0.97_AlSiN_3_:0.03Eu^2+^@Eu_2_O_3_, Ca_0.97_AlSiN_3_:0.03Eu^2+^@EuN, Ca_0.97_AlSiN_3_:0.03Eu^2+^@EuB_6_ as well as YAG:0.06Ce^3+^, respectively.

It is obvious that CASN:Eu^2+^@EuB_6_ has a preferable thermal stability than others. The increased thermal stability is attributable to the partial substitution of Eu–O (Eu–N) bonds by more covalent Eu–B ones that leads to a higher structural rigidity, which has already been observed in carbon-doped nitride phosphors [40,41,42]. Therefore, using nano-EuB_6_ as a raw material in nitride luminescence materials can improve the luminescence performance as well as the thermal stability.

### 3.5. The Fabrication of W-LEDs Device

**Figure 9:**
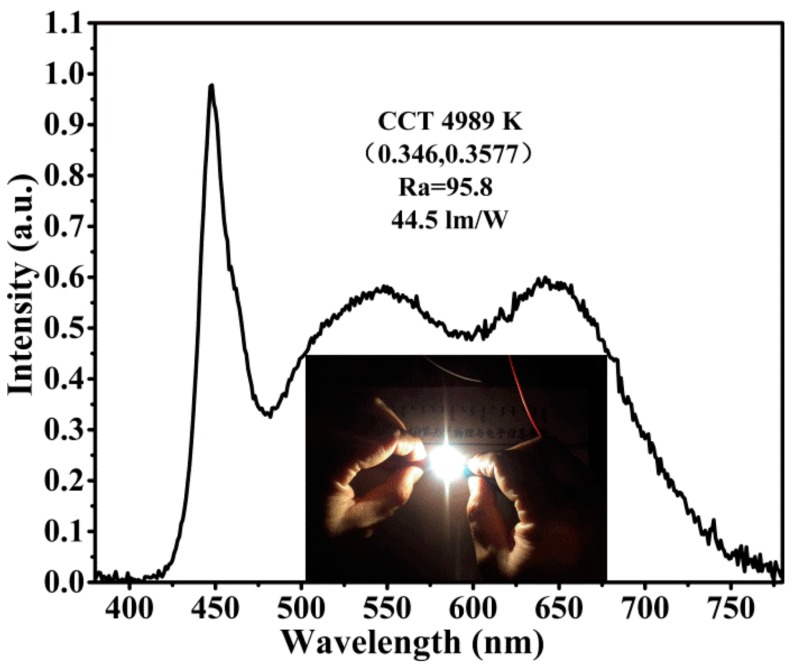
The normalized PL spectrum of a W-LED using a blue-LED chip (455 nm) and G3537 and Ca_0.97_AlSiN_3_:0.03Eu^2+^@EuB_6_ phosphors.

To demonstrate the potential application of the as-synthesized Ca_0.97_AlSiN_3_:0.03Eu^2+^@EuB_6_ red nitrides phosphor, a prototype of W-LEDs was fabricated by combining a 455 nm blue LED chip with a mixture of commercial green phosphor G3537 (produced by Dalian Luming) and the as-synthesized red phosphor Ca_0.97_AlSiN_3_:0.03Eu^2+^@EuB_6_. The normalized photoluminescence (PL) spectrum of the as-fabricated W-LEDs is shown in Figure 9. The corresponding CRI, CCT, luminous efficiency and CIE chromaticity coordinates were determined to be 95.8, 4989 K, 44.5 lm/W and (0.3460, 0.3577), respectively. The value of CRI is higher than that of traditional W-LED production made by combining yellow phosphor YAG:Ce^3+^ with blue LED chip (Ra ≈ 75). The W-LED packaging results indicate that the as-synthesized Ca_0.97_AlSiN_3_:0.03Eu^2+^@EuB_6_ is a potential candidate as a red phosphor for W-LEDs based on blue LEDs.

## 4. Conclusions

In this paper, three series of CASN:Eu^2+^ red nitride phosphors, using Eu_2_O_3_, EuN and EuB_6_ as raw materials, were successfully synthesized by the alloy precursor normal pressure nitridation. The morphologies, crystal phases, compositions, XPS spectra as well as luminescence properties were investigated in detail. Interrelated Analysis of XPS indicates that the Eu^2+^ content in CASN:Eu^2+^@EuB_6_ is significantly higher than in CASN:Eu^2+^@Eu_2_O_3_, thus it causes a stronger emission intensity of CASN:Eu^2+^@EuB_6_ than others. Furthermore, CASN:Eu^2+^@EuB_6_ has a preferable thermal stability is attributable to the partial substitution of Eu–O (Eu–N) bonds by more covalent Eu-B ones that leads to a higher structural rigidity. Consequently, nano-EuB_6_ doped red nitride phosphor has the potential for application in high-power pc-LEDs and using nano-EuB_6_ as raw material by the alloy precursor normal pressure nitridation method possesses the high advantage of relative low reaction temperature, cheap raw materials and simple processing.

## Figures and Tables

**Table 1:** EDS analytical data of CaSi alloys, Ca_0.94_AlSiN_3_:0.06Eu^2+^@Eu_2_O_3_, Ca_0.94_AlSiN_3_:0.06Eu^2+^@EuN and Ca_0.94_AlSiN_3_:0.06Eu^2+^@EuB_6_.

Element (wt %)	B	N	O	Al	Si	Ca	Eu
CaSi alloys	——	——	4.28	——	59.39	36.33	——
Ca_0.94_AlSiN_3_:0.06Eu^2+^@Eu_2_O_3_	——	21.52	6.19	18.05	28.53	22.53	3.26
Ca_0.94_AlSiN_3_:0.06Eu^2+^@EuN	——	36.33	2.78	18.04	23.83	15.76	3.25
Ca_0.94_AlSiN_3_:0.06Eu^2+^@EuB_6_	5.17	25.56	1.95	18.27	26.49	19.32	3.27

**Table 2:** Intensity ratios of Eu^2+^/Eu^3+^ in Ca_0.94_AlSiN_3_:0.06Eu^2+^@Eu_2_O_3_, Ca_0.94_AlSiN_3_:0.06Eu^2+^@EuN and Ca_0.94_AlSiN_3_:0.06Eu^2+^@EuB_6_.

XPS Measurements	Intensity of Eu^2+^	Intensity of Eu^3+^	Eu^2+^/Eu^3+^
Ca_0.94_AlSiN_3_:0.06Eu^2+^@EuN(3*d_3/2_*)	0.8847	0.9333	9.48/10
Ca_0.94_AlSiN_3_:0.06Eu^2+^@EuN(3*d_5/2_*)	0.8596	0.9854	8.72/10
Ca_0.94_AlSiN_3_:0.06Eu^2+^@EuB_6_(3*d_3/2_*)	0.9166	0.9633	9.52/10
Ca_0.94_AlSiN_3_:0.06Eu^2+^@EuB_6_(3*d_5/2_*)	0.8817	1. 0000	8.82/10
